# Safety climate perceived by pre-hospital emergency care personnel—an international cross-sectional study

**DOI:** 10.3389/fpubh.2023.1192315

**Published:** 2023-07-17

**Authors:** Justyna Kosydar-Bochenek, Dorota Religa, Małgorzata Knap, Marcin Czop, Bartosz Knap, Wioletta Mędrzycka-Dąbrowska, Sabina Krupa

**Affiliations:** ^1^Institute of Health Sciences, College of Medical Sciences of the University of Rzeszow, Rzeszów, Poland; ^2^Division for Clinical Geriatrics, Department of Neurobiology, Care Sciences and Society (NVS), Karolinska Institute, Stockholm, Sweden; ^3^Institute of Health Sciences, Collegium Medicum of the Jan Kochanowski University of Kielce, Kielce, Poland; ^4^Department of Clinical Genetics, Medical University of Lublin, Lublin, Poland; ^5^Doctoral School, Medical University of Lublin, Lublin, Poland; ^6^Chair and Department of Experimental and Clinical Pharmacology, Medical University of Lublin, Lublin, Poland,; ^7^Department of Anesthesiology Nursing and Intensive Care, Faculty of Health Sciences, Medical University of Gdańsk, Gdańsk, Poland

**Keywords:** safety climate, safety culture, safety attitudes questionnaire, emergency medical services, pre-hospital emergency care, paramedic, nurse

## Abstract

**Introduction:**

Improving patient safety is one of the most critical components of modern healthcare. Emergency medical services (EMS) are, by nature, a challenging environment for ensuring patient safety. It is fast-paced, physically dangerous, and highly stressful, requiring rapid decision-making and action. This can create risks not only for patients but also for employees. We assessed variations in perceptions of safety culture in prehospital emergency care among an international sample of paramedics and nurses.

**Methods:**

The Emergency Medical Services Safety Attitudes Questionnaire (EMS-SAQ) was used for the study. The instrument measures six domains of safety culture in the workplace: teamwork climate, job satisfaction, safety climate, working conditions, stress recognition, and perceptions of management. A total of 1,128 EMS from 9 countries participated in this study.

**Results:**

Safety Climate was 81.32/100 (SD 6.90), Teamwork Climate 84.14/100 (SD 8.74), Perceptions of Management 76.30/100 (SD 10.54), Stress Recognition 89.86/100 (SD 5.70), Working Conditions 81.07/100 (SD 9.75), and Job Satisfaction 70.71/100 (SD 7.21). There was significant variation in safety culture scores across countries for teamwork climate (TWC), working conditions (WC), and job satisfaction (JS). Among the individual variables (age, gender, level of education, and work experience), variations in safety culture scores were unaffected by age, gender, or work experience. Organizational characteristics: employment status and position type were linked to significant variations in safety culture domain scores.

**Conclusion:**

Participants’ perceptions of the patient safety climate were not particularly satisfactory, confirming that there is still a need to develop a culture of patient safety in prehospital emergency care.

## 1. Introduction

One of the most important aspects of contemporary healthcare is increasing patient safety. Patient safety is viewed as the outcome of group efforts to protect patients from harm by preventing medical errors or avoidable adverse occurrences (1).

Pre-hospital emergency care is an inherently challenging setting and an area where patient safety is difficult to ensure due to the fast-paced and unpredictable nature of this environment (2). The prehospital emergency medical services (EMS) main goal is to provide individuals with life-saving care when they are most in need of it. EMS is essential for public safety and the functioning of the healthcare system (3). Patient safety is challenging in this sector due to the combination of patients with complex high-acuity conditions, patient turnover, lack of control over workload, and communication challenges. An emergency medical services working environment is difficult to control, and multidisciplinary teamwork requires frequent handovers with the risk of communication breakdowns and potential loss of vital information (4). A bad safety culture can manifest itself in back injuries, medication errors, and other adverse consequences for the provider and the patient, as well as in misdiagnosis of patient symptoms and signs and deviations from standard protocols of treatment (5, 6). Interventions of paramedics and nurses in pre-hospital care often involve procedures that, when performed incorrectly or at the wrong time, can cause serious harm to patients (1). Pre-hospital emergency care has other unique characteristics: these services are smaller than other healthcare organization services, they comprise only a few professional groups, their professional work is always performed in small teams (usually typically two or three providers per team), and there is a defined hierarchy within these teams. These characteristics generate a work environment in which personnel plays a major role; therefore, special attention to organizational culture and teamwork is needed in pre-hospital emergency care (7).

Safety climate is a measure of frontline healthcare workers’ shared perceptions, behaviors, beliefs, and attitudes toward the organization’s safety culture. Safety climate scores are also associated with the frequency of errors and adverse events in the healthcare setting (8). Despite the critical role of pre-hospital emergency care, these services are often a neglected component of the healthcare system, including patient safety. Pre-hospital emergency care is relatively new as a separate discipline, and many of its devices, procedures, and settings are based on practices inherited from hospital settings and are similar to those used in intensive care units (7). It should be noted, however, that many of these inherited in-hospital practices are not suited to the unique work environment of pre-hospital care, and this incongruity may ultimately affect the health and safety of both the medical staff and their patients (9).

Safety culture in emergency medical services has been the subject of studies, e.g., in the United States, Canada, Finland, Sweden, Portugal, Brazil (2, 5–7, 10). Previous investigations have studied organizational safety culture in skilled nursing facilities, ambulatory care, nursing wards, intensive care units (ICUs), and hospital emergency department (11–14). Publications on safety climate in pre-hospital emergency care are scarce and this area requires in-depth research to better understand the magnitude of the problem and threats to patient safety and to guide interventions (15).

Workplace attitudes, culture, and beliefs can influence the safety of patient care. The study aimed at identifying which factors may influence the perception of safety culture in an international EMS sample.

## 2. Materials and methods

### 2.1. Study design

We carried out a cross-sectional investigation. The purpose of this study is to assess the attitudes of emergency medical personnel toward patient safety.

The aim of the study is to answer the following research questions:

- What are the attitudes of emergency medical personnel toward patient safety factors?
- What are the variations in attitudes toward safety in the countries studied?
- What are the relationships between individual and organizational characteristics and safety attitudes?

### 2.2. Characteristics of the research tool

Data was collected using The Emergency Medical Services Safety Attitudes Questionnaire (EMS-SAQ). Patterson and colleagues created the EMS-SAQ by adapting a validated safety culture tool, the Safety Attitudes Questionnaire (SAQ), and the Intensive Care Unit Safety Attitude Questionnaire (ICU-SAQ) to the EMS environment (6). As a validated safety culture survey, the SAQ can serve as a standard measurement tool to compare safety culture levels across organizations. However, this tool can also measure an organization’s safety culture level (7).

EMS-SAQ is designed to assess safety culture in pre-hospital emergency medical services. It consists of six domains and has 30 questions that are rated on a five-point Likert scale. The questions characterize six areas of safety culture:

- Safety Climate (SC; seven questions),
- Teamwork Climate (TWC; six questions),
- Job Satisfaction (JS; five questions),
- Perceptions of Management (PM; four questions),
- Working Conditions (WC; four questions), and
- Stress Recognition (SR; four questions).

The participants respond to each item using a five-point Likert scale (1 = strongly disagree, 2 = slightly disagree, 3 = neutral, 4 = slightly agree, 5 = strongly agree). Two questions (9 and 17) are coded backward to correspond to the positive valence of the remaining items (2). According to Sexton et al., the responses were converted to a 100-point scale measurement as follows: Disagree strongly = 0, Disagree slightly = 25, Neutral = 50, Agree slightly = 75, and Agree strongly = 100 (11). The EMS-SAQ scores were analyzed in two ways (16). Domain item scores were summed and divided by the total number of domain items to calculate the mean domain score. The percentage of positive responses was calculated by identifying the percentage of respondents with an average score of 75 or higher for each domain.

SC is the perception of a proactive and strong commitment to safety in the organization. TWC is the perceived level of staff members’ collaboration. SR is the recognition of how stressors influence performance. The PM is the managerial action’s approval. WC is the perceived quality of the work setting and logistic support (equipment, staff, etc.), while JS is the degree of satisfaction with the work experience (4, 6).

The EMS-SAQ has shown good psychometric characteristics. The validation studies confirm feasibility with acceptable internal consistency, high response rate, and good model fit. Assessment of the validity and reliability of the structures of the 6 domains utilizing CFA demonstrated acceptable model fit and validity (CSDFr = 1.2; CFI = 0.95; and NNFI = 0.92). In comparison to prior adaptations of the SAQ6, there was acceptable internal consistency (reliability) for five of the six scales: SC (a = 0.83), TWC (a = 0.80), SR (a = 0.71), WC (a = 0.71), and JS (a = 0.88). Internal consistency for PM was 0.65 (6). Subsequent surveys of an EMS staff sample showed good internal consistency (Cronbach’s alpha for SC, alpha = 0.82; TWC, alpha = 0.83; PM, alpha = 0.68; JS, alpha = 0.8; WC, alpha = 0.75, and SR, alpha = 0.78). Instrument validity testing confirmed the presence of a six-domain structure and good model fit properties: RMSEA = 0.04, CFI = 0.97, NNFI = 0.95 (2).

### 2.3. Data collection, setting, and procedure

Data was collected from the 15th of August to the 30th of September 2022 through a web-based survey that was shared with participants. Prior to commencing the study, the authors obtained consent from the creators of the survey tool for its use. The survey was targeted toward full-time or part-time paramedics and nurses who provide pre-hospital emergency care. The questionnaire was distributed through the websites of international medical personnel associations and via email to their members. Besides, the link was posted on the organization’s social media profiles and shared in groups whose members are mainly EMS professionals or have a particular interest in the EMS. An online pool managed by a single-country research group is the only way to conduct an international survey. Also, it was quick, simple, and practical to gather and evaluate the data using the online survey. The survey was conducted in English. The study’s goals were briefly explained to the respondents before they completed the survey. Respondents answered a brief demographic survey at the end. No identifiable personal information about the responders was required for the purpose of guaranteeing anonymity. The participants gave their informed consent to take part in the investigation by filling out the questionnaire, although they were free to change their minds at any time. The full questionnaire took between 10 and 15 min to complete. It was made clear to respondents that participation was optional and anonymous, and that all replies would be kept private and not be made available to management.

### 2.4. Inclusion and exclusion criteria

Inclusion criteria: individuals working as paramedics or nurses in prehospital emergency care; working in prehospital emergency care for at least 4 weeks, professionally active; knowledge of the English language at least at the Intermediate level.

Exclusion criteria: persons who work as paramedics or other medical personnel in a hospital emergency department (ED); lack of knowledge of the English language; working in the pre-hospital ambulance service for less than a month, with no professional activity.

### 2.5. Ethical considerations

The University of Rzeszow’s Bioethics Committee gave its approval to this study (KBE No. 2022/013). The authors adhered to the Declaration of Helsinki’s guidelines (17).

### 2.6. Statistics

Statistical analyses were done with Statistica software (v13.3, StatSoft, Poland). Data expressed on a quantitative scale was presented as mean and standard deviation (SD). Qualitative data was presented as a number and percentage of the sample. Depending on the number of groups compared and the Shapiro–Wilk test result, the following tests were employed: the Mann–Whitney test and the Kruskal-Wallis test (with subsequent *post hoc* test). Results were considered statistically significant when *p* < 0.05.

## 3. Results

### 3.1. Participants

The questionnaire was completed by 1.134 people. 1,128 questionnaires were utilized in the analysis (6 questionnaires were not complete). 767 women (68%) and 361 men (32%) took part in the study. The most frequent age stratum was 31–40 years (52.13%) and 18–30 years (34.75%). Respondents came from different countries around the world: Italy (4.88%), Portugal (7.36%), England (42.46%), China (7.36%), Netherlands (15.16%), Germany (2.93%), France (5.32%), Finland (6.12%), and Japan (8.42%). Healthcare workers with 6–10 years of experience constituted the largest group (44.24%), with a large group also consisting of people with less than 5 years of experience (35.55%). The most frequent stratum for total years of experience in the current EMS agency was over 11 years (42.29%). Respondents were mostly EMT–paramedics (33.69%) and prehospital RNs (23.23%). More than one-half of the respondents (58.78%) were full-time employees and most of them had higher education and an associate’s or bachelor’s degree (65.43%). Table 1 details the characteristics of the participating emergency medical services.

**Table 1:** Participants’ characteristics (*n* = 1,128).

	*N*	%
**Gender**		
Female	767	68.00
Male	361	32.00
**Age**		
18–30 years	392	34.75
31–40 years	588	52.13
41–50 years	99	8.78
>50 years	49	4.34
**Country**		
Italy	55	4.88
Portugal	83	7.36
England	479	42.46
China	83	7.36
Netherland	171	15.16
Germany	33	2.93
France	60	5.32
Finland	69	6.12
Japan	95	8.42
**Total experience in EMS**		
≤5 years	401	35.55
6–10 years	499	44.24
11–15 years	64	5.67
16–20 years	85	7.54
>20 years	79	7.00
**Years at current EMS**		
≤5 years	297	26.33
6–10 years	354	31.38
>11 years	477	42.29
**Position type**		
EMT-basic	167	14.80
EMT-intermediate	97	8.60
EMT-paramedic	380	33.69
Prehospital RN	262	23.23
Other	222	19.68
**Employment status**		
Career full-time	663	58.78
Career part-time	440	39.01
Volunteer	25	2.22
**Education**		
Some high school, high school graduate, or GED	263	23.32
Some college	127	11.26
College (graduate)	393	34.84
College (AD or bachelor’s)	345	30.59

### 3.2. Results of emergency medical services safety attitudes questionnaire in the research group

**Figure 1:**
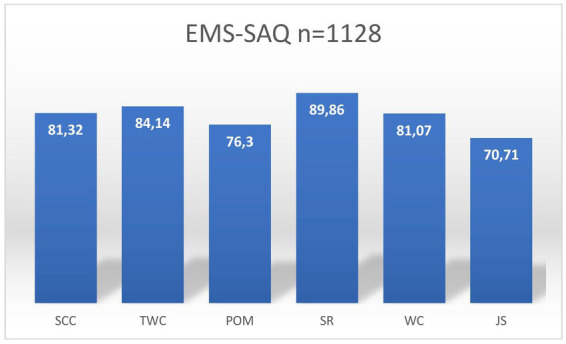
Total mean scores for each safety culture domains.

The main results were the survey scores for each of the safety domains: (1) Safety Climate (SC), (2) teamwork climate (TWC), (3) perceptions of management (PM) (4) stress recognition (SR), (5) working conditions (WC), and (6) job satisfaction (JS). Safety Climate was 81.32 (SD 6.90), Teamwork Climate 84.14 (SD 8.74), Perceptions of Management 76.30 (SD 10.54), Stress Recognition 89.86 (SD 5.70), Working Conditions 81.07 (SD 9.75) and Job Satisfaction 70.71 (SD 7.21). The results are presented in Figure 1.

For the dimensions of the investigation (SC, TC, PM, SR, WC, and JS), means and SDs were calculated for gender, age, level of education, work experience, working level, job status, and position type. The results are presented in Table 2.

**Table 2:** Variation in safety culture domain scores between the respondent’s characteristic.

	SCC		TWC		POM		SR		WC		JS	
	Mean	SD	Mean	SD	Mean	SD	Mean	SD	Mean	SD	Mean	SD
**Gender**												
Male	80.98	7.03	84.85	8.36	75.81	10.66	89.56	5.82	80.75	9.96	70.21	7.19
Female	81.49	6.84	83.81	8.90	76.53	10.49	90.00	5.64	81.22	9.64	70.95	7.21
**Age**												
18–30 years	80.58	7.32	84.05	8.59	76.16	10.94	90.07	5.43	80.87	10.43	70.33	6.85
31–40 years	81.65	6.67	84.14	8.93	75.87	10.24	89.59	5.80	81.19	9.34	71.12	7.19
41–50 years	81.42	6.47	84.51	8.42	78.79	10.60	90.91	5.81	81.50	9.57	69.04	8.26
>50 years	83.09	6.63	84.10	8.49	77.55	10.28	89.29	6.25	80.36	9.38	72.24	7.43
**Country**												
Italy	81.36	7.49	86.14^a^	6.62	76.48	11.53	87.39	5.83	79.09^a^	8.94	70.36^ab^	7.00
Portugal	80.64	7.79	85.79^a^	6.95	77.03	10.92	90.51	5.11	71.69^b^	8.47	66.87^a^	8.07
England	81.40	6.74	86.20^a^	6.79	76.30	10.30	89.90	5.67	83.26^a^	8.16	71.26^b^	7.08
China	81.33	7.59	85.94^a^	6.66	76.28	11.33	90.44	5.38	83.28^a^	8.57	70.60^ab^	6.69
Netherland	81.33	6.40	86.01^a^	5.99	76.43	10.59	89.51	5.41	82.75^a^	7.92	70.94^b^	6.86
Germany	81.93	7.03	85.10^a^	5.71	75.19	9.69	90.15	6.06	85.42^a^	9.07	71.52^ab^	7.23
France	81.43	6.57	63.89^b^	7.26	76.35	9.97	90.10	6.12	83.13^a^	8.08	70.75^ab^	7.24
Finland	81.16	7.27	73.01^b^	5.96	75.54	11.78	90.40	6.99	65.13^b^	9.49	70.80^ab^	8.21
Japan	81.32	6.92	86.71^a^	6.72	76.25	10.09	90.00	5.42	83.16^a^	8.11	70.84^b^	6.71
**Experience**												
≤5 years	80.74	7.28	84.03	8.58	76.18	10.99	90.06	5.44	81.00	10.41	70.45	6.78
6–10 years	81.58	6.66	83.83	9.15	75.80	10.11	89.72	5.77	80.80	9.47	71.22	7.38
11–15 years	82.03	6.94	85.48	8.00	75.98	10.34	88.48	5.48	82.03	8.73	69.30	7.28
16–20 years	81.01	6.55	83.87	8.23	78.75	10.88	91.03	5.90	81.91	9.55	69.76	7.63
>20 years	82.41	6.61	85.81	7.85	77.69	10.43	89.64	6.32	81.41	9.06	71.01	7.57
**Years at current EMS**												
≤5 years	80.78	6.69	84.05	9.00	75.65	10.59	89.88	6.01	80.58	9.95	70.17	7.03
6–10 years	81.53	7.12	83.66	9.08	75.71	11.07	89.76	5.48	81.34	9.28	71.12	6.78
>11 years	81.51	6.86	84.55	8.30	77.15	10.06	89.92	5.67	81.17	9.96	70.75	7.61
**Position type**												
Prehospital RN	81.23	6.71	83.57	8.12	74.93^b^	10.63	90.22	5.69	80.30	9.83	70.21	7.25
EMT-paramedic	81.17	7.16	84.59	8.51	78.67^a^	10.86	89.65	5.65	81.00	9.80	71.03	7.73
EMT-basic	81.65	6.23	82.86	10.06	72.87^b^	9.70	89.82	6.19	81.62	8.78	70.48	6.45
EMT-intermediate	81.96	7.64	84.28	8.87	79.51^a^	9.23	89.24	5.97	80.61	10.27	71.80	7.33
**Employment status**												
Career full-time	82.25^b^	6.56	83.84	8.73	75.08^b^	10.39	89.72	5.83	80.17^b^	9.76	70.54	7.29
Career part-time	80.11^a^	7.17	84.67	8.79	78.30^a^	10.49	90.04	5.55	82.47^a^	9.64	71.07	7.05
Volunteer	78.14^a^	7.02	82.83	7.73	73.75^b^	10.21	90.25	4.80	80.25^b^	8.59	69.20	7.86
**Education**												
Some high school, high school graduate, or GED	81.36	6.91	84.52	8.52	76.95	10.32	89.90	5.88	80.44	10.61	70.72	7.26
Some college	81.24	6.84	84.06	9.26	77.56	10.09	90.31	5.66	80.71	9.35	70.55	7.57
College (graduate)	81.19	6.76	84.41	8.98	75.25	11.34	89.73	5.54	81.63	9.50	70.76	7.24
College (AD or bachelor’s)	81.48	7.10	83.56	8.43	76.54	9.85	89.82	5.76	81.03	9.47	70.71	7.03

Means not sharing the same letter in the column are significantly different at *p* < 0.01.

Among the individual variables (gender, age, education level, and work experience), variations in safety culture scores were unaffected by age, gender, or work experience.

Organizational characteristics: employment status and job type were linked to significant variations in safety culture domain scores. All position-type groups had the highest mean scores on the SR subscale. The lowest scores were for JS and PM. The mean MP score was lower for Prehospital RN and EMT-basic than the mean score for the other position types (Table 2). Employment status had a significant impact on the views of EMS staff in the domains of SC, PM, and WC. The mean SC scores were highest among respondents with full-time careers. However, respondents working part-time had higher scores in the domains of PM and WC.

The country of origin of EMS personnel had a significant impact on the scores in the TC, WC, and JS domains. EMS personnel from France (mean 63.89) and Finland (mean 73.01) had significantly lower scores in the TW domain than personnel from other countries. EMS personnel working in Finland (mean 65.13) and Portugal (mean 71.69) had lower scores for WC than staff working in other countries. JS scores in pairwise comparisons were significantly lower for EMS personnel working in Portugal (mean 66.87).

The findings in Table 3 represent the percentage of positive responses (PPR) in each domain of the questionnaire for each group. A value higher than or equal to 75 (≥ 75) was taken as a cut-off for a positive response. The PPR for Safety Climate varied according to the number of years of activity in the EMS and the employment status. The PPR for Teamwork Climate varied from country to country. The lowest results were obtained in Finland (79.47) and France (61.39). The PPR for Perceptions of Management differed by age and was the highest among EMT–intermediates (83.51) and part-time respondents (81.76). The PPR for Working Conditions varied from country to country. The lowest results were recorded in Portugal (68.98) and Finland (65.22).

**Table 3:** Variations in the percentage of positive responses across individual characteristics.

	SCC		TWC		POM		SR		WC		JS	
	Mean	SD	Mean	SD	Mean	SD	Mean	SD	Mean	SD	Mean	SD
**Gender**												
Male	84.69	11.42	91.64	11.99	76.11	20.30	99.03	4.83	85.18	16.62	73.46	11.62
Female	84.54	10.85	90.68	12.42	77.35	20.58	99.05	4.77	85.20	16.39	74.24	11.36
**Age**												
18–30 years	83.20	11.80	90.73	12.18	75.32^a^	21.49	99.04	4.80	84.50	17.17	73.06	10.93
31–40 years	85.25	10.65	91.07	12.50	76.83^ab^	19.68	99.02	4.85	85.67	15.64	74.69	11.22
41–50 years	85.43	9.99	91.41	11.51	82.58^b^	19.38	99.24	4.31	84.85	17.43	71.92	13.97
>50 years	86.01	10.31	91.16	12.31	80.10^ab^	22.23	98.98	5.00	85.71	18.40	77.14	11.55
**Country**												
Italy	84.42	12.38	93.94^a^	10.33	77.27	23.21	99.09	4.72	79.09^a^	20.28	73.45	10.92
Portugal	83.48	11.04	93.17^a^	9.75	78.31	19.82	99.10	4.69	68.98	14.91	68.92	14.73
England	84.73	10.87	93.56^a^	9.32	76.93	20.14	99.06	4.76	88.52^a^	14.69	74.61	11.06
China	84.68	11.97	93.57^a^	10.36	76.81	21.66	98.80	5.39	87.95^a^	14.80	73.98	11.15
Netherland	84.63	10.62	93.27^a^	10.16	77.19	20.26	99.12	4.61	88.89^a^	14.38	74.15	11.42
Germany	85.28	11.56	93.43^a^	9.26	75.00	19.76	98.48	6.06	88.64^a^	15.42	75.15	10.04
France	84.52	9.94	61.39^b^	8.40	77.08	20.73	99.17	4.53	88.75^a^	13.36	73.67	11.34
Finland	84.89	11.20	79.47^c^	9.54	76.09	21.62	98.91	5.14	65.22^b^	12.29	74.78	12.20
Japan	84.36	11.62	94.21^a^	8.69	76.58	20.57	99.21	4.40	88.16^a^	14.05	74.53	9.87
**Total experience in EMS**												
≤5 years	83.43	11.78	90.65	12.16	75.44^a^	21.47	99.00	4.90	84.54	17.26	73.37	10.90
6–10 years	85.17	10.49	90.71	12.74	76.55^ab^	19.63	99.15	4.4	85.02	15.84	74.75	11.43
11–15 years	84.82	11.35	91.93	11.88	78.52^ab^	20.83	98.44	6.0	88.67	14.73	72.50	10.98
16–20 years	85.21	10.21	91.18	11.38	82.94^b^	18.98	99.71	2.71	85.59	16.54	72.94	13.35
>20 years	85.90	10.85	93.46	11.14	79.43^ab^	21.09	98.42	6.13	86.39	17.36	74.68	12.28
**Years at current EMS**												
≤5 years	82.40^a^	11.11	90.57	13.03	76.77	20.83	99.07	4.73	84.85	16.11	72.05^a^	11.57
6–10 years	85.23^b^	10.85	90.25	12.35	76.13	20.24	99.22	4.34	85.10	16.47	75.14^b^	10.49
>11 years	85.47^b^	10.95	91.79	11.72	77.67	20.48	98.90	5.13	85.48	16.69	74.34^ab^	11.91
**Position type**												
Prehospital RN	84.68	10.82	90.46	11.86	75.00^bc^	20.53	99.33	4.04	84.92	15.99	73.13	12.32
EMT-paramedic	84.06	11.39	91.32	12.07	79.47^ac^	19.72	98.82	5.32	85.20	17.03	74.37	11.48
EMT-basic	85.63	9.47	90.72	13.14	73.05^b^	21.25	98.80	5.36	85.18	14.30	73.77	10.27
EMT-intermediate	85.13	10.50	90.03	12.19	83.51^a^	18.00	99.74	2.54	84.79	17.88	75.46	11.73
Other	84.36	11.96	91.67	12.56	75.00^bc^	21.14	98.99	4.94	85.70	17.01	73.87	11.03
**Employment status**												
Career full-time	86.15^a^	11.04	91.15	12.41	73.79^a^	20.85	98.76	5.44	83.14^a^	16.99	73.42	11.59
Career part-time	82.50^b^	10.49	90.80	12.19	81.76^b^	18.97	99.43	3.73	88.24^b^	15.36	75.05	10.97
Volunteer	80.00^ab^	13.04	90.00	10.76	76.00^ab^	21.02	100.00	0.00	86.00^ab^	12.67	70.40	14.28
**Education**												
Some high school, high school graduate, or GED	84.25	10.96	91.70	11.91	78.71	18.74	99.05	4.79	83.17	17.05	74.22	11.46
Some college	84.03	11.61	90.55	12.35	79.13	19.61	99.41	3.81	84.84	16.41	73.54	11.79
College (graduate)	84.77	10.66	91.26	12.99	75.19	21.39	99.05	4.80	85.81	16.19	74.45	11.46
College (AD or bachelor’s)	84.84	11.31	90.29	11.71	76.81	20.93	98.91	5.11	86.16	16.25	73.45	11.31

Means not sharing the same letter in the column are significantly different at *p* < 0.05.

## 4. Discussion

In this survey sample, we observed variations in perceptions of safety culture in EMS across countries. The great variation in the safety culture of the workplace is not surprising, as the EMS work environment involves numerous risks to the safety of patients and providers. Potential factors underlying differences in culture include differences in regional practices, economic resources, and leadership structures and styles. In addition, there exists no common international mechanism for the classification and reporting of errors, medical malpractice, and adverse events in EMS (2).

The EMS-SAQ Questionnaire is an underused tool for assessing the safety climate in pre-hospital care. To date, several studies have been conducted using this tool (2, 4, 6). Patterson et al. conducted a study using the EMS-SAQ Questionnaire in the United States and Canada. Safety culture scores varied considerably from one emergency medical care organization to another: Safety climate averaged 74.5, Teamwork Climate mean 71.2, Perceptions of Management mean 67.2, Job Satisfaction mean 75.4, Working Conditions mean 66.9, and Stress Recognition mean 55.1. Furthermore, air medical care organizations showed a tendency to score higher in all domains of safety culture, perhaps because the culture of safety originated in the aviation industry. Lower scores in safety culture were linked to an increase in yearly patient contacts. Scores in the safety climate domain were not linked to other characteristics of the individuals or the EMS agency (2).

A study using the Finnish version of the EMS-SAQ showed that the overall mean scores for each safety culture domain were considered non-positive (mean score < 75); safety climate 60.12, teamwork climate 60.92, management perception 56.31, stress recognition 64.55, working conditions 53.43 and job satisfaction 70.36. In addition, higher education was related to lower job satisfaction and teamwork climate within the individual characteristics. The study found that all organization-related characteristics resulted in at least one significant safety culture score variation. Work area had a significant effect (*p* < 0.05) on five of the six domains of safety culture. As per the findings, organization-based characteristics are more likely to influence safety attitudes than individual characteristics (4).

The emergency departments (ED) of hospitals embody a working environment that is akin to that of emergency medical services and is also associated with the saving of human life and health. The ED work environment is also fast, physically unsafe, and very stressful, requiring quick decision-making and action.

Rigobello et al. revealed that the safety climate in the ED was unsatisfactory, which could have negative clinical consequences for patients. They found that the dynamic environment in the ED requires distinct approaches to improving patient safety (9). Verbeek Van Noord et al. identified dimensions of safety culture in the ED including teamwork, frequency of event reporting, communication openness, learning from errors, management support, and overall perceptions of patient safety (18). Alshyyab et al. claim that safety culture varies across healthcare settings due to the interplay between cultural and social factors (1). The main factors influencing safety culture in the ED were human factors, managerial factors, organizational, and environmental factors. Human factors include the perception of the employees toward patient safety and the procedures and systems pursued to prevent errors. Managerial factors include leadership and support from hospital management and supervisors. Organizational and environmental factors, such as error reporting also influenced patient safety.

A study using another safety climate assessment tool, the Victorian Managed Insurance Authority Safety Climate Survey was conducted among doctors and nurses in an Australian emergency department. Nurses rated the commitment of the organization to patient safety higher than physicians in all attitudinal domains (*p* < 0.05), except for stress recognition. Both groups believe that fatigue, increased workload, and stress recognition negatively affect patient safety. In addition, there was a significant trend toward lower ratings of safety climate related to participants’ level of clinical competence and experience in all domains except stress recognition (*p* < 0.05). At the same time, emergency department physicians and nurses perceived no strong organizational commitment to patient safety in the Australian emergency department (8).

The authors of a study conducted in Turkey came to some interesting conclusions. The investigation was designed to determine emergency department nurses’ attitudes toward patient safety. The survey was conducted with ED nurses. Data were collected with the use of tools like the “Patient Safety Attitude Scale” and the “Information Questionnaire.” The study found that ED nurses’ attitudes toward patient safety were average and unrelated to gender, age, education level, ED certification, ED experience, patient safety training, nursing experience, and nurses’ perceptions of patient safety with respect to self-efficacy, ED quality certification or hospital quality certification. Nurses’ attitudes toward patient safety were compared by gender, age, education level, marital status, and ED experience, and no substantial differences were found. A significant difference was observed between age groups and the subdimension “defining stress” of the Patient Safety Attitude Scale. In contrast, ED nurses’ certification status in emergency care, quality training, patient safety training, and hospital or ED certification status in quality showed no statistically significant difference (13, 14).

### 4.1. Study limitations

The survey presented here has several limitations. The EMS-SAQ tool relies heavily on self-reported behavior. The information obtained may be biased and not correctly reflect the current situation. Positive responses may be biased. Staff perceptions may change over time and be affected by daily events in a changing work environment. The questionnaire was answered mostly by EMS providers from European countries therefore the results are probably not completely generalizable. Differences in safety culture scores across EMS agencies in each geographic area and respondent characteristics warrant further study. There are differences in the performance of emergency medical systems in every studied country which may translate into differences in perceptions of safety climate. In addition, since the goal is to create a safe emergency medical system and reduce the number of adverse medical events, the relationship between patient safety in EMS and patient outcomes should also be investigated.

## 5. Conclusion

The EMS-SAQ serves as a valuable tool to reliably measure and evaluate the safety climate in pre-hospital emergency care. Workplace safety culture varied significantly in this sample of EMS pre-hospital emergency workers. The variation in safety culture scores in pre-hospital emergency care across countries within a different geographic region, and the variation in the characteristics of respondents, requires more in-depth research. Organizational-related characteristics, such as position type and employment status, were more likely to have an impact on safety attitudes than individual-related characteristics. Therefore, it is suggested that EMS organizations undertake safety culture development at the organizational level. The EMS-SAQ can provide insights into prehospital safety. Little research has been done on patient safety in the EMS setting, hence it is poorly understood. The culture of patient safety in European healthcare still must be developed. Pre-hospital emergency care can provide a safer environment through a genuine commitment to improving a safety culture that leverages insights, internal strengths, and behaviors specific to staff knowledge and experience. Such commitment can positively impact the effectiveness of efforts to minimize patient safety risks and improve job satisfaction and staff efficiency.

## 6. Implications for practice

Pre-hospital emergency care services can thus apply the EMS-SAQ as a tool for monitoring changes in staff attitudes and indicating specific safety domains that require intervention and focus. The challenge facing emergency department management is understanding the complex nature of patient safety, the factors influencing it, and the strategies to be employed to create a more positive work culture and climate to improve patient safety. Improving the safety culture in the EMS should be a national priority in any emergency medical system.

## Data availability statement

The raw data supporting the conclusions of this article will be made available by the authors, without undue reservation.

## Ethics statement

The studies involving human participants were reviewed and approved by Bioethics Committee of the University of Rzeszow (KBE No. 2022/013). The patients/participants provided their written informed consent to participate in this study.

## Author contributions

JK-B and SK: conceptualization and writing—original draft preparation. WM-D, DR, and SK: methodology. MC and BK: software. MC and MK: formal analysis and data curation. MK: resources. WM-D, SK, and JK-B: writing—review and editing. WM-D and JK-B: visualization. DR: supervision. JK-B: project administration. All authors contributed to the article and approved the submitted version.

## Conflict of interest

The authors declare that the research was conducted in the absence of any commercial or financial relationships that could be construed as a potential conflict of interest.

## Publisher’s note

All claims expressed in this article are solely those of the authors and do not necessarily represent those of their affiliated organizations, or those of the publisher, the editors and the reviewers. Any product that may be evaluated in this article, or claim that may be made by its manufacturer, is not guaranteed or endorsed by the publisher.
